# Nanobubbles as ultrasound contrast agent for facilitating small cell lung cancer imaging

**DOI:** 10.18632/oncotarget.18155

**Published:** 2017-05-24

**Authors:** Jin-Ping Wang, Xiao-Lin Zhou, Ji-Ping Yan, Rong-Qin Zheng, Wei Wang

**Affiliations:** ^1^ Key Laboratory of Chemical Biology and Molecular Engineering of Ministry of Education, Institute of Biotechnology, Shanxi University, Taiyuan, Shanxi, China; ^2^ Department of Ultrasound, Shanxi Provincial People's Hospital, Taiyuan, Shanxi, China; ^3^ Radiation Medicine Laboratory, Institute of Radiation and Environmental Medicine, China Institute for Radiation Protection, Taiyuan, Shanxi, China; ^4^ Department of Ultrasound, The Third Affiliated Hospital, Sun Yat-Sen University, Guangzhou, Guangdong, China

**Keywords:** small cell lung cancer, nanobubbles, contrast-enhanced ultrasound, peak time, area under the curve and peak intensity

## Abstract

**Background:**

This study is to investigate whether liposome-loaded nanobubbles (NBs) have the potentials to carry anti-pro-gastrin releasing peptide (proGRP) antibody and enhance ultrasound imaging of small cell lung cancer (SCLC).

**Methods:**

NBs were loaded with an antibody against SCLC (H446 cell line). A nude mouse model of SCLC tumor was established by a subcutaneous injection of tumor cell suspension in the dorsal skin. Images for contrast-enhanced ultrasound (CEUS) of xenograft tumors in the model were obtained through an intravenous injection of blank and targeting NBs.

**Results:**

The targeted NBs showed a high binding affinity (90.2 ± 3.24%) of the H446 cells in vitro as compared to the blank NBs that have no affinity of the cells. In process of tumor imaging, no mice died of the NB application. CEUS imaging of the targeted NBs manifested significant increases in half-peak time, area under the curve and peak intensity as compared to the blank NBs. In the model of SCLC, treatment with targeting NBs resulted in a large amount of fluorescent dye accumulated in the tumor tissue but not the liver tissue.

**Conclusion:**

Our results indicate that NBs can carry antibody traveling to the SCLC cells, whereas application of NBs is safe and reliable in serving as ultrasound contrast agents for improving SCLC imaging.

## INTRODUCTION

Small cell lung cancer (SCLC) is an aggressive subtype of lung cancer and accounts for approximately 20% of all lung cancer cases [1]. More than two-thirds of the cancer patients have the early development of widespread metastases at diagnosis with the median survival time of 12-16 months [2]. Gastrin-releasing peptide (proGRP), a member of the bombesin family of peptides, has been shown to have mitogenic activity in SCLC, and to be produced by SCLC in an autocrine fashion [3]. The peptide as a useful tumor marker has been applied for clinical practice in early diagnosis of SCLC [4].

NB technology is currently being incorporated into a massive range of applications in the medical field. Since NBs carrying antibodies or ligands can recognize the specific molecules on target cells. Targeting NBs as a promising tool has been applied for helping SCLC ultrasonographic imaging and improving therapeutic efficacy of the tumor [5–7]. Due to the fact that nano-scale bubbles with a large diameter are difficult to penetrate into the extra-vascular space of cancerous tissue through the endothelial cell gaps of 780 nm [4], the NBs with a diameter < 700 nm would be crucial in observing extravascular imaging of tumor tissue.

This study was to explore efficacy of targeting NBs in improving echogenicity of SCLC tissue. Our results indicate that NBs can carry pro-GRP antibody to identify the SCLC cells in the xenograft tumor model, whereas the NBs serve as ultrasound contrast agents for facilitating SCLC imaging.

## MATERIALS AND METHODS

### Preparation of liposomes

Dil-loaded liposomes were prepared using a thin-film hydration-sonication method. Briefly, all phospholipids including 18mg dipalmitoyl phosphatidylcholine (DPPC), 1.0 mg 1,2-distearoyl-sn-glycero-3-phosphoethanolamine-polyethylene-glycol 2000-maleimide (DSPE-PEG2000-maleimide), 1.0 mg diphenylphosphoryl azide (DPPA) and 1.0 mg 1,1’-dioctadecyl-3,3,3’,3’-tetramethylindocarbocyanine perchlorate (Dil) were dissolved in 4 ml of chloroform and then transferred into a 9-cm culture dish to form a thin phospholipid film by natural evaporation. The film was hydrated with 4 ml of hydration liquid consisting of 10% glycerol (v/v) and 2 mg/ml Pluronic F-68, and then maintained in a shaking incubator at 60 °C for 1 h to form the Dil-loaded liposomes. The liposomal suspension was transferred into a 50 ml centrifuge tube.

### Preparation of anti-pro-GRP antibody

BALB/c mice were immunized with recombinant pro-GRP. Mouse spleen cells were harvested and fused with mouse myeloma Sp2/0 cells. The monoclonal anti-pro-GRP antibody was collected from the ascites after the antibody-labeled cells were injected into the abdominal cavity of mice. To make the single-chain anti-pro-GRP antibody, solution A was prepared with 10 μg anti-pro-GRP antibody and 10 μl of 0.5 M EDTA dissolved in 50 μl of PBS. Solution B was prepared in 500 μl of PBS containing 60 mg of mercaptoethylamine and 10 μl of 0.5 M EDTA. To obtain a high purified production of the single-chain anti-pro-GRP antibody, the mixture of solution A and B was incubated at 37°C for 90 min and then was centrifuged with 500 μl of PBS containing 10 μl of 0.5 M EDTA three times (3000 rpm x 8 min) at 4°C.

### Preparation of targeting nanobubbles

The single-chain anti-pro-GRP antibody was mixed with 500 μl of liposome at 4°C overnight. The antibody was resuspended in 0.5 ml of PBS after it was centrifuged with PBS three times to remove the free antibody. 10ml of C_3_F_8_ gas (Research Institute of Physical and Chemical Engineering of Nuclear Industry, Tianjin, China) was slowly injected into the solution containing the antibody using a syringe. The solution was rigorously shaken for 45 seconds. After standing still, the foam layer was removed from the top of the solution and the milky white suspension was used as the targeting NBs. Blank NBs were prepared by mixing the solution containing the liposomes and C_3_F_8_ gas in same procedures.

### Identification of nanobubbles

Briefly, the dried solutions were incubated with 50 mM Tris [2-carboxyethyl]phosphine (TCEP, Sigma) in 25 mM NH_4_HCO_3_ at 56°Cfor 1 hour to reductively cleave the disulfide bonds of proteins. The resulting sulfhydryl functional groups were alkylated with 100 mM iodoacetamide (IAA, Amersham) in 25 mM NH_4_HCO_3_ at room temperature for 0.5 hour in the dark. Subsequently, the proteins were digested with 20 ng/μl porcine trypsin (modified proteomics grade, Sigma) at 37°C overnight. The mixture of the resulting tryptic peptides was extracted twice from the gel pieces with 5% trifluoroacetic acid (TFA, Fluka) in a 50% ACN solution. The pooled extracts were evaporated in a vacuum centrifuge (Labconco, Kansas, MO), and resuspended in 0.1% methanoic acid (Sigma) prior to the LC separation and MS detection. In-house database was constructed with the FASTA protein sequences downloaded from uniprot mapping (http://www.uniprot.org) containing all proteins from mouse. For each experiment, MS/MS spectra were extracted from the raw data files by extractmsn program in the Proteome Discoverer 2.0 (ThermoFinnigan, San Jose, CA).

### Characterization of nanobubbles

The morphology of NBs was examined using a transmission electron microscope (TEM) (JEM-1011, JEOL, Inc., Peabody, MA, USA). The mean diameter and polydispersity of NBs were measured using photon correlation spectroscopy (90 Plus particle sizer, Brookhaven Instruments Corp). Measurements were performed at 25°C with a laser wavelength of 660 nm at an angle of 90°. Bubble size was measured by diluting a sample formulation 1:1000, with PBS at pH 7.4. The bubble size was reported as the number average. The zeta potential of NBs was determined by using a Zeta Plus Analyzer (90Plus/BI-MAS, Brookhaven, Holtsville, NY, USA). Measurements were performed at 25°C and each sample was run using 7 repetitions and the average was reported.

### Cell culture

SCLC cells (H446 cell line) and lung cancer cells (NCI-H292 cell line) from China Institute for Radiation Protection were cultured in RPMI-1640 medium supplemented with 10% fetal bovine serum and 1% penicillin-streptomycin in a 5% CO_2_ incubator at 37°C. The cells were passaged with 0.25% trypsin every 3–4 days. The cells used in our study can grow exponentially at a constant rate.

### Cellular immunofluorescence assay

The cells were cultured in a 6-well plate covered with coverslips at a density of 1.5 × 10^4^ cell/well and the plate was placed in the incubator overnight. After the medium was removed, cells on coverslips were washed with PBS and fixed with 4% paraformaldehyde for 20 minutes at room temperature, After the cells were treated with 1% bovine serum albumin (BSA) for 30 minutes at room temperature, they were incubated with anti-pro-GRP antibody (1:100 dilution) at 4°C overnight. The cells were incubated with Alexa Fluor 555 conjugated goat anti-mouse secondary antibody (1:2000 dilution) at 37°C for 1 hour in the dark and stained with DAPI for 3 minutes after washing with PBS. The cells on coverslips were sealed and observed under a laser confocal microscope (Olympus fv 1000). PBS was applied to replace primary antibody in the control group.

### Binding ability of nanobubbles to the H446 cells

SCLC cells were seeded in a 6-well plate containing coverslips with 1.5 × 10^4^ cells in each well. After overnight incubation, cells on coverslips were fixed in 4% paraformaldehyde for 20 minutes at room temperature, and blocked with 1% BSA at room temperature for 30 minutes after washing with PBS, and placed in a wet box at 4°C overnight, 30 μl targeting NBs, blank NBs and PBS were added to the cells in the climbing tablets respectively, and incubated at 37°C for 1 hour in the dark. The cells were stained with DAPI for 3 minutes after washing with PBS. Then the sections were sealed for further observation under confocal laser scanning microscope (Olympus FV 1000, Olympus, Tokyo, Japan). Twenty microscopic fields were randomly selected with100 cells examined in each microscopic field. Cells adhered to more than 4 NBs were regarded positive cells. An adhesion rate of NBs to the cells was expressed as the percentage of positive cells in total cells.

### Mouse model of xenograft tumor

Specific pathogen-free, male BALB/c mice (4-5 weeks) weighing about 23g were kept at 25°C in a 12:12 h light-dark cycle and followed by free access to both food and water supplies. H446 cells were centrifuged and resuspended in DMEM with a density of 1×10^7^ cells/ml after digested with 0.25% trypsin solution. Each mouse received a subcutaneous injection of 2.0 × 10^6^ cells in the dorsal skin. All procedures in this study were reviewed and approved by the Ethics Committee of Shanxi University and Shanxi Provincial People's Hospital.

### CEUS of the xenograft

Mice were anesthetized with 100 ml of 5% chloral hydrate and immobilized. The ultrasound probe of LOGIQ-E9 diagnosis apparatus (GE, USA) was placed on the mouse chest and xenograft tumor. Contrast-enhanced ultrasound (CEUS) for blood flow condition in the areas and the size of the tumor were evaluated by B-mode and color Doppler flow imaging (CDFI). Briefly, 30 ul of blank NBs (5μg/ml) were first injected into each mouse via the tail vein. After CEUS of the NBs was performed with a 9 MHz 9L-D transducer, normal saline was applied for the animal to empty ultrasound NBs into blood vessels. Next, 30 ul of targeting NBs was injected into the same mouse at 20 minutes apart and CEUS of the targeted NBs were obtained to compare effects of NBs on enhanced ultrasound imaging of tumor. During the imaging process, these parameters were controlled at an output power of 10%, a 54 dB dynamic range, mechanical index 0.15, gain amplifier 90%, and focus depth 2-3 cm. A 5 mm^2^ region of interest (ROI) was placed in the tumor where the concentration of contrast medium was highest. The time-intensity curve was obtained using the Gamma variate and the following parameters were calculated, including area under the curve, time to peak, arrival time, peak intensity, and half-peak time.

### Histologic examination

30μl of the Dil labeled NBs were injected into nude mice via the tail vein. The mice were immobilized on the bench. The procedure for heart perfusion of the animals was performed by injection of 0.9% saline. Briefly, an infusion needle was inserted into left ventricular and the right atrial appendage was cut in a small incision as a blood outflow pathway. In order to well remove the residual contrast agent from circulatory system of mice, perfusion was stopped until color of mouse liver became pale. The specimens from the tumor tissue and the liver tissue were prepared for frozen sections. The specimens were stained with Hoechst (1: 1000). The retention of NBs in both tissues was observed under laser scanning confocal microscope (LSCM). DyLight488-labeled goat secondary antibody to mouse IgG (1: 1000), green color of fluorescence, was added to the sections. The cells were incubated at 37° C for 1 hour in the dark and stained with DAPI for 3 minutes. The retention of NBs in tumor and liver tissues was observed under LSCM.

### Statistical analysis

Data were expressed as Mean ± Standard deviation (SD). Student's paired t-test was used to compare measurements regarding the effects of targeting NBs on the tumor cells stained by antibodies. Statistical analyses were performed using Statistical Package for the Social Science (SPSS, version 21.0). A P value of < 0.05 was considered significant.

## RESULTS

### Characterization of the nanobubbles

**Figure 1:**
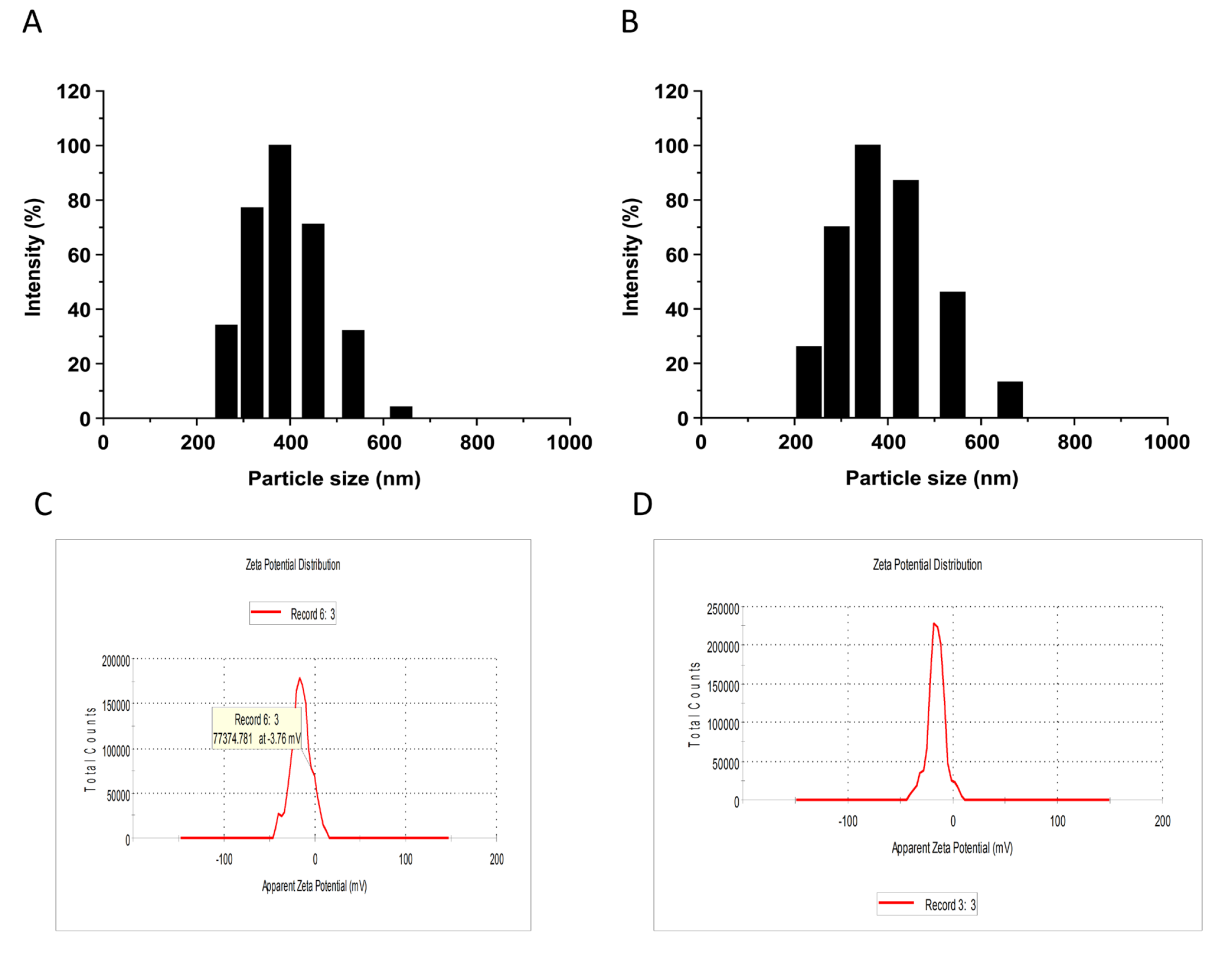
Histograms of the particle size of the targeted nanobubbles (**A**) and the blank nanobubbles (**B**). Zeta potential of the targeted nanobubbles (**C**) and the blank nanobubbles (**D**).

**Figure 2:**
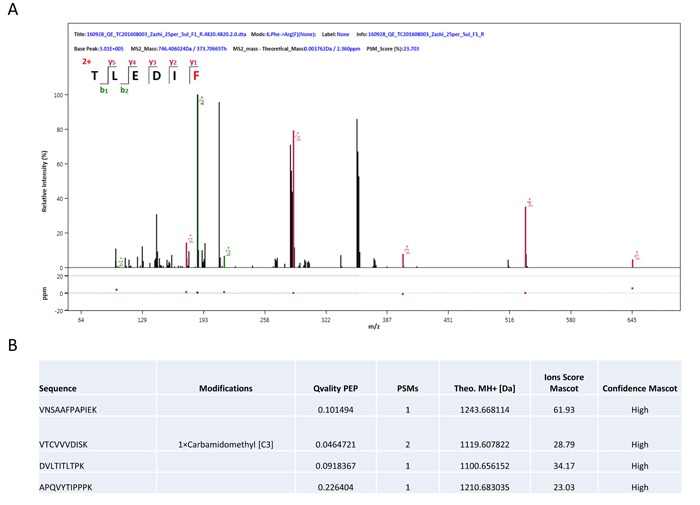
Identification of nanobubbles (**A**) Peptide sequences were detected by tandem mass spectrometry based on the bands obtained from protein gel electrophoresis. Only b and y fragment ions were accounted. (**B**) MS/MS spectra matched to IgG1. All of the four peptides came from Igh and Ighg1.

Freshly generated NBs were prepared for each experiment. Appearance of liposome NBs in suspension were milk white. NBs are spherical particles with the similar sizes viewed under phase contrast microscope at a high magnification (400x). No microscopic evidence of NB aggregation was observed. Diameters of targeting and blank NBs were shown as 378.1 ± 50.7 nm and 356.9 ± 45.7 nm with no significant difference found in the size between them (Figure 1A, 1B). Polydispersity index (P.I.) of the targeting and blank NBs were 0.154 ± 0.044 and 0.161 ± 0.040. Zeta potential of NBs, which is indicative of NB's stability, was examined with the results shown as -15.2 ± 2.64 mv and -17.6 ± 3.21 mv in targeting and non-targeting NBs, respectively (Figure 1C, 1D). There were no statistical differences found in the zeta potential between them. Tandem mass spectrometry was used for quantification of anti-pro-GRP on the surface of NBs and the results displayed the antibody load (Figure 2A, 2B), indicating the capacity of the ligand as single chain to recognize the target.

### Specificity of the targeted nanobubbles towards small cell lung cancer

**Figure 3:**
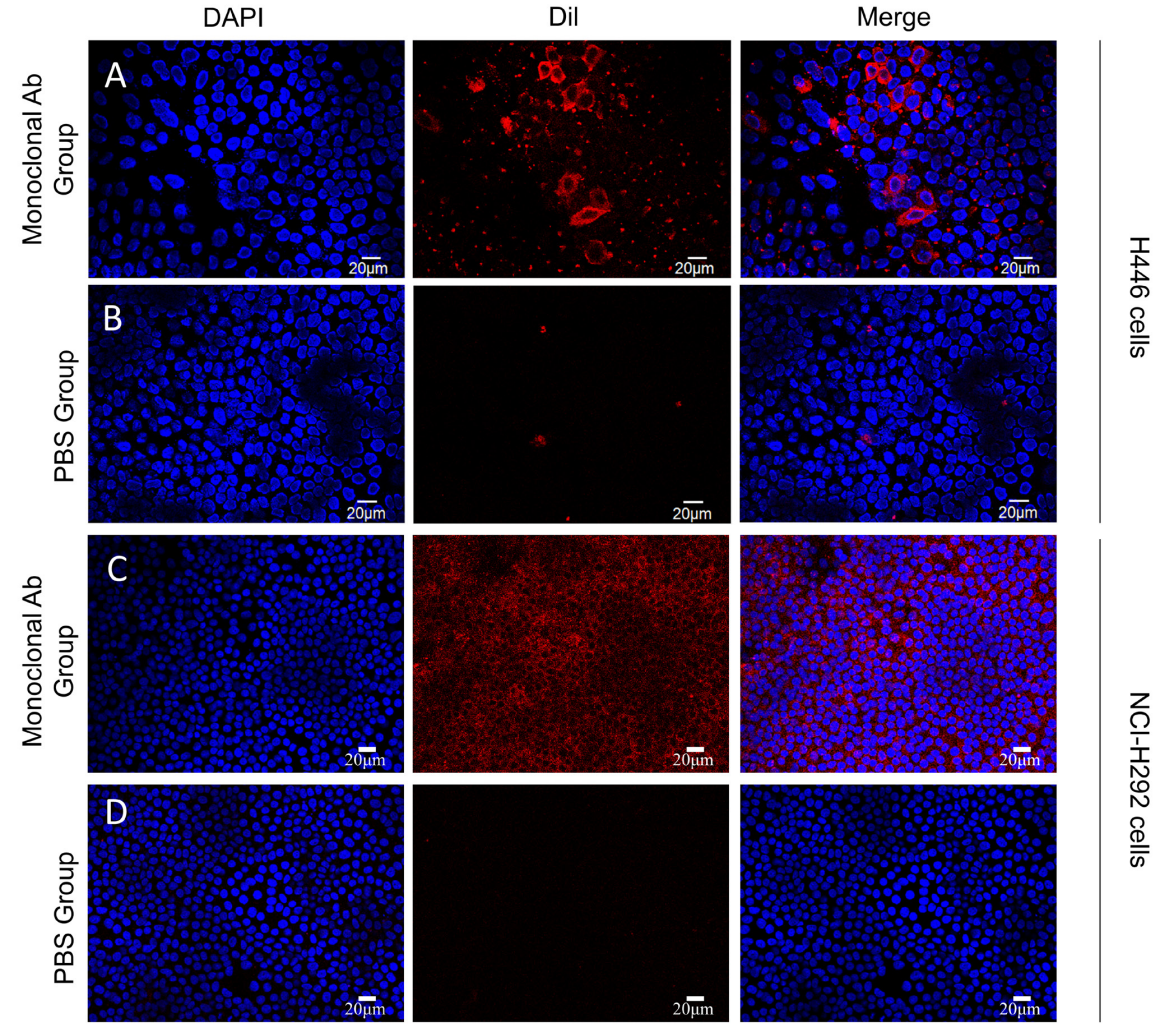
Cellular immunology assays were employed for observing the positive response of antibody to SCLC H446 (**A**) and NCI-H292 cells (**C**). PBS was used as a control for SCLC H446 (**B**) and NCI-H292 cells (**D**). Images were obtained under a light microscope at 40x magnification.

Immunological assay was performed to test specificity of targeted nanobubbles to detect small cell lung cancer H446 cells and the normal human lung tissue cell line NCI-H292. The H446 cells showed strong red fluorescence around the nucleus in the monoclonal antibody, but not when PBS was used (Figure 3A, 3B), or in the NCI-H292 cells (Figure 3C, 3D). The high specificity of targeting bubbles is perhaps due to the pro-gastrin releasing peptide (GRP) expressed within the SCLC cells. This might also explain the higher persistence of targeting over non-targeting nanobubbles in that the process is antigen expression based. It is a pertinent question about what happens to the non-targeting bubble, which will be actively pursued in future research endeavors.

### *In vitro* targeting: binding of nanobubbles and cells

**Figure 4:**
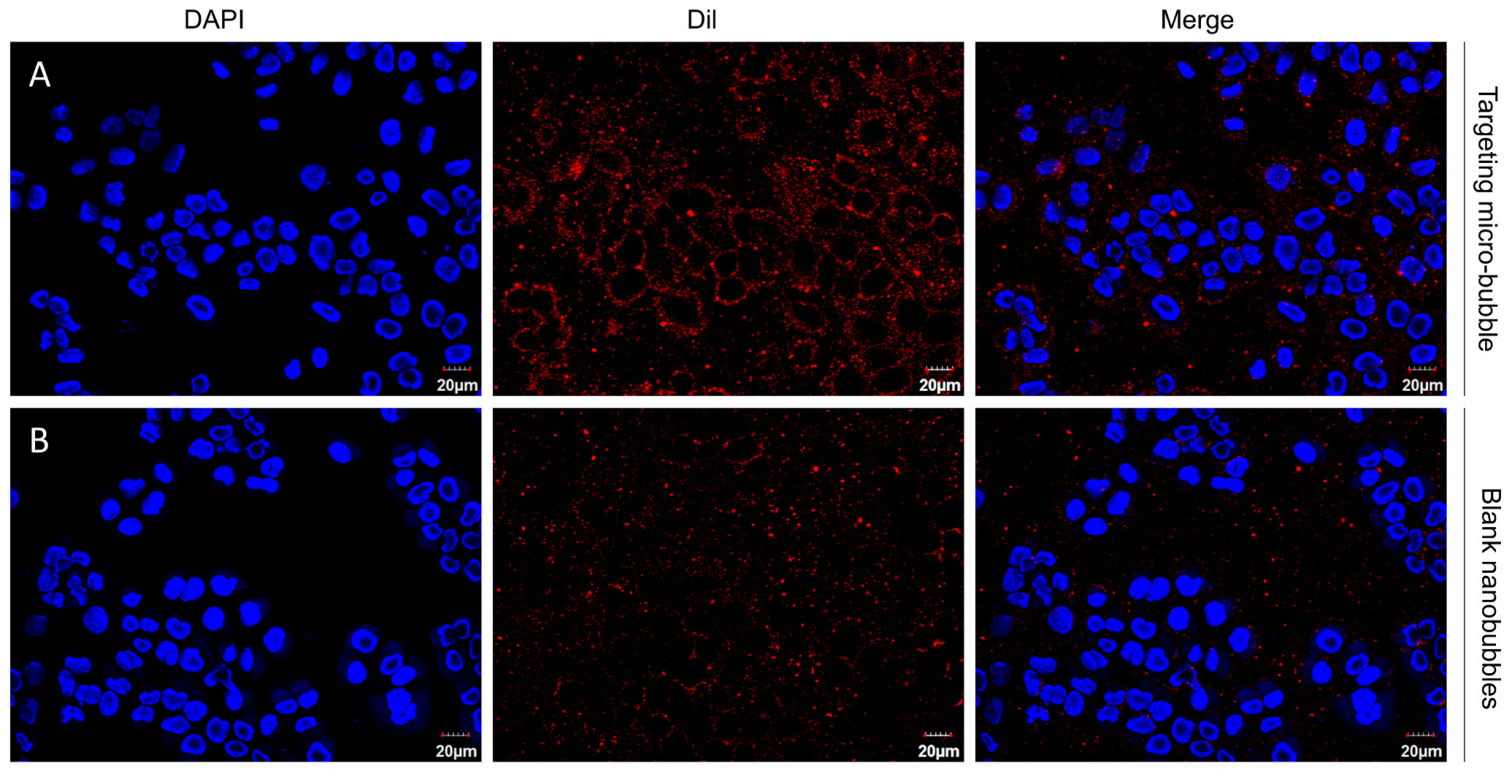
Binding of targeted NBs (**A**) and blank NBs (**B**) in vitro to pro-GRP-positive H446 cells. Images were obtained under a light microscope at 40x magnification.

After adding H446 cells to target nanobubbles and blank nanobubble incubator, the red fluorescence probe aggregation of H446 cells in blue-stained H446 cells was observed in 1000 target nanobubbles and was 90.2 ± 3.24%. (Figure 4A) No obvious red fluorescent probe aggregation was observed around the nucleus of the small cell lung cancer H446 cell in the blank nanobubble group (Figure 4B), indicating that the SCLC cells around the targeted nanobubbles, but not blank nanobubbles, only exhibited high adhesion.

### CEUS with nanobubbles

**Figure 5:**
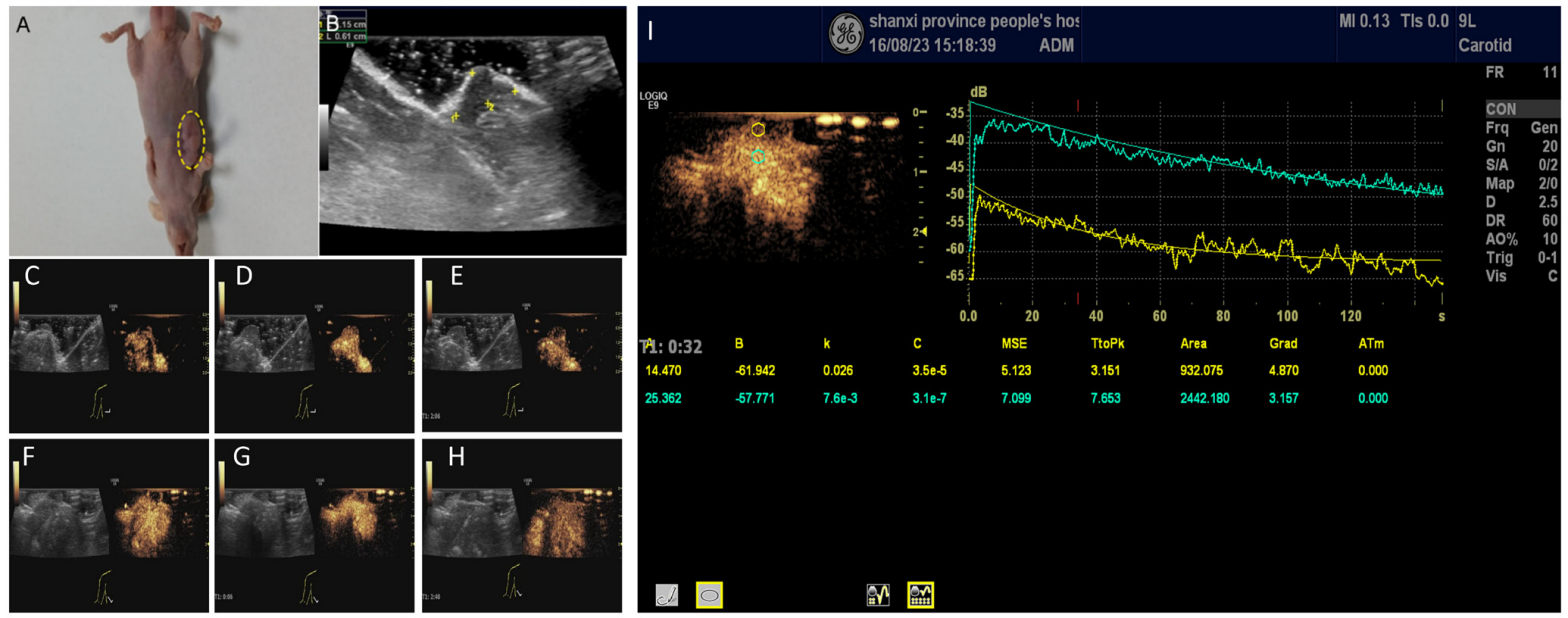
Contrast-enhanced ultrasonography of xenograft tumor model A profile of SCLC xenograft tumor (**A**) Measurement of the tumor size showed a diameter of 1.18 ± 0.12 cm (**B,** **C**-**E**) Early-stage of the contrast-enhanced ultrasonography of the tumor (C), peak intensity of the contrast-enhanced ultrasonography (D), and late-stage of contrast-enhanced ultrasonography (E) in targeting nanobubbles. Yellow dotted circles denote the tumor. Images in C, D, and E include B-mode and contrast images. (**F**-**G**) Same as C-E, except for in non-targeting blank nanobubbles. (**I**) Comparison of the time-intensity curves between the cancerous (yellow) and non-cancerous tissue (green).

The inoculated H446 cells grew to a tumor of 1.0 cm after 4 weeks in 19 mice (Figure 5A, 5B). One mouse showed no xenograft tumor after 4 weeks. No animal died during the experiment. *In vivo* imaging of non-targeted and targeted NBs using the same experimental animal was done as internal-control with the same animal to reduce tumor heterogeneity and other experimental errors. Experimental program was to first observe the imaging effect of non-targeted NBs, then look at the imaging effect of targeting NBs. Since non-targeted NBs do not carry antibodies, there was no significant effect on blood circulation and tumor microenvironment in the experimental animals; and there was a 20-min intermittent period (wash period) which was sufficient for NBs to be completely cleared *in vivo* before injection of NBs. CDFI showed dotted blood flow signal in the xenograft tumor (Figure 5C, 5D) with targeted nanobubbles, but not with non-targeting nanobubbles (Figure 5F-5H).

CEUS with the targeted nanobubbles showed significantly higher peak intensity, area under the curve, and half-peak time in comparison with the blank nanobubbles (Table 1). There was no significant difference in time to arrival and time to peak between the targeted and the blank nanobubbles. Similarly, each parameter was significantly higher in tumor tissue compared to liver tissue (Figure 5I, Table 2), proving that the targeted nanobubbles penetrate the neovascular vessels of tumors and enter the tissue space.

**Table 1:** Comparison of targeting NBs and blank NBs in SCLC imaging (Mean ± SD)

	Imaging start time (s)	Peak time (s)	Peak intensity (db)	AUC	Half-peak time (s)
Targeting nano bubble	3.15±0.13	4.27±0.54	25.06±0.83*	899.81±8.13*	172.79±8.96*
Blank nano bubble	2.97±0.10	8.91±0.76	23.03±0.13	333.87±9.87	140.63±7.92

Note: paired t test, (*P<0.05)

**Table 2:** Comparison of targeted NBs in ultrasound-enhanced imaging of SCLC and liver (Mean ± SD)

	Imaging starting time (s)	Peak time (s)	Peak intensity (db)	AUC	Half-peak time (s)
Tumor	3.15±0.13*	4.27 ±0.54*	22.06±0.83*	899.81±8.13*	172.79±8.96*
Liver	7.08±0.10	8.91±0.76	33.07±0.13	2442.87±9.87	145.23±6.81

Note: paired t test, (*P<0.05)

### Immunohistochemistry analysis

**Figure 6:**
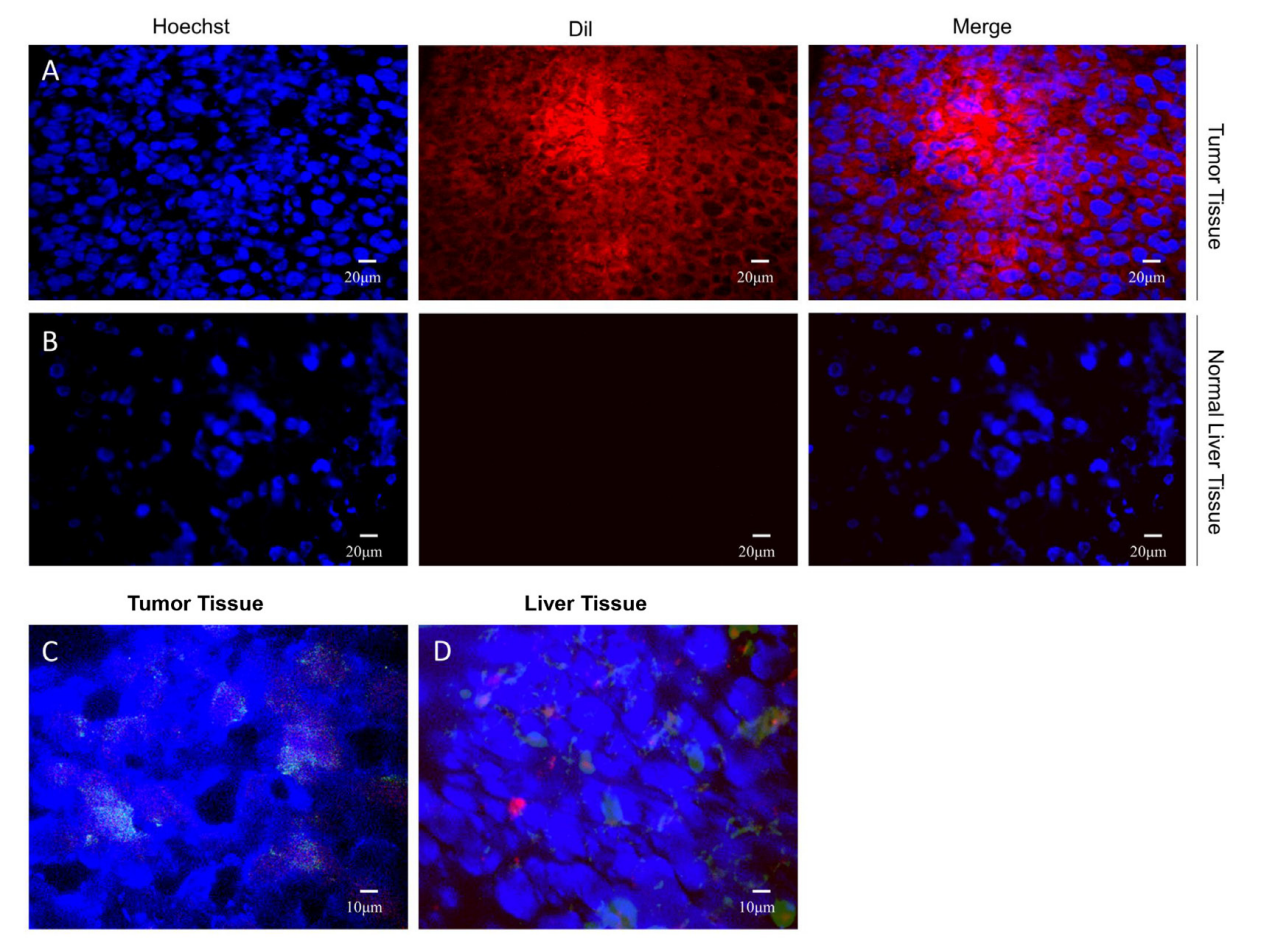
Dil-labeled NBs enter only the tumor tissue DilImmunofluorescence of xenograft tumor specimens shows Dil-NBs around the nucleus in tumor tissue (**A**), but not in the normal liver tissue (**B**) Green fluorescence around the nucleus of blue-stained tumor tissue (**C**), whereas no obvious fluorescence around the nucleus in the liver tissue (**D**).

Experimental animals were sacrificed after the examination of CEUS in identifying effects of NBs on the tumor tissue was performed. Frozen sections were obtained from the tumor and liver tissue. In contrast, laser confocal microscopic observation of the tumor but not liver showed red fluorescent color gathered around the cell nucleus (Figure 6A, 6B), indicating that this diameter of nano-particles can not only carry antibody arriving to SCLC tissue but also enter into the interstitial space of the tissues via blood vessel walls. Since blood vessels of normal liver are continuous with tight endothelial cell-cell junctions, most NBs stayed in liver tissue. DyLight488-labeled goat anti-mouse IgG antibody was added to the frozen sections. The targeting bubbles in blood flow to the tumor and liver tissues were imaged with laser confocal microscope. The results indicated that green fluorescent staining was visible outside of the blood vessels around the blue-stained tumor tissue. However, the staining was not detected in the liver tissue (Figure 6C, 6D), demonstrating that the Dil-NBs entered the tumor tissue but not the liver tissue.

## DISCUSSION

Molecular targeted technique as a promising tool has been applied for patients with SCLC for years [8–10]. A small particle size of a targeting bubble is a basic requirement for ultrasound contrast-enhanced agents that penetrate tumor blood vessel pores to allow for targeted imaging and therapy [11]. In this work, we generated a lipid NBs contrast-enhanced ultrasound agent in the size range of 356.9 ± 45.7 nm. Even though the polydispersity index (P.I.) of the targeting and blank NBs (0.154 ± 0.044 and 0.161 ± 0.040, respectively) were within acceptable limits, the slightly high values were indicative that the process can be improved further to obtain greater homogeneity among both the targeting and blank NBs. The result indicates that the targeted NBs would have the ability to pass through the gaps between the endothelial cells in the tumor vascular system to enter the tissue space [4, 12]. The use of 1,2-distearoyl-sn-glycero-3-phosphoethanolamine-polyethylene-glycol 2000-maleimide (DSPE-PEG2000-maleimide), which has a negative charge, as one of the shell components was done to ensure that the nanobubbles had an overall negative charge which will maintain their stability in water. In addition, the overall negative charge will maintain the nanobubbles as dispersible and biocompatible in water. Zeta potential was analyzed to measure the stability of NBs [13]. The result showed that there was no difference in distribution of the electric potential between blank and targeting NBs, suggesting that the nanoscale bubbles were of the same stability in data acquisition of contrast-enhanced imaging of tissues. *In vitro* studies showed that the pro-GRP-loaded NBs can attach to SCLC cells with high affinity of the cells, indicating that targeting NBs not only have the ability to carry antibody but also are able to deliver the antibody to recognize the cancer cells. Our findings supported the reports that applications of NBs produce an optimal contrast enhancement effect in ultrasound imaging of tumors and benefit cancer therapy [14–16]. In this study, the disulfide bonds in the anti-pro-GRP antibody structure were broken by mercaptoethylamine to reduce the molecular weight with the exposed sulfhydryl groups in the Fc region of the antibody. The single-chain antibody was bound to the NBs through strong thioether bonds formed on the bubble surface by the thiol reaction. Given no significant difference found in the particle size between the targeted and the blank NBs (Figure 1A, 1B), it led us to conclude that that the association with the primary antibody did not alter the particle size of NBs. Incubation with DyLight 488-labeled secondary antibody resulted in showing green fluorescence around the SCLC cells, indicating that the anti-pro-GRP antibody traveled to SCLC cells through the NB-carrier effect.

To evaluate SCLC imaging enhancement ability of NBs, *in vivo* observation was performed on the tumor-carrying nude mice. No animals died during the experiments. Upon caudal vein injection, the feasibility of using CEUS for characterizing blood flow was examined in the cancer xenograft model. The results manifested significant changes in half-peak time, AUC, and peak intensity calculated from the tumor and liver tissues treated with and without targeting NBs. These findings provided evidence that intravenous injection of targeting NBs can penetrate into the tissue space accessed via the tumor vessels since solid tumors typically have leaky blood vessels and nanoparticles tend to seep out of the vessel openings and congregate around tumors [17, 18]. Though a concentration of the targeted NBs in this study was stable with a longer acquisition time for diagnostic imaging of the SCLC tissue, immunohistochemical examination did not show signs of the NBs stayed in the tumor tissue. This might be related to an inadequate amount of the antibody loaded on the NBs. Since our data showed that NBs are capable of improving ultrasound contrast imaging for tumors and organs, it's conceivable that combined ultrasound imaging with nanoparticle-based targeted chemotherapy would have important applications in diagnosis and treatment of cancers.

In conclusion, this study indicates that NBs with a small size can not only carry antibody arriving to SCLC cells but also serve as ultrasound contrast agents for facilitating SCLC imaging.

## Abbreviations

NBs: nanobubbles
proGRP: anti-pro-gastrin releasing peptide
SCLC: small cell lung cancer
CEUS: contrast-enhanced ultrasound
DPPC: dipalmitoyl phosphatidylcholine
DPPA: diphenylphosphoryl azide
Dil: 1,1’-dioctadecyl-3,3,3’,3’-tetramethylindocarbocyanine perchlorate
TEM: transmission electron microscope
BSA: bovine serum albumin
CDFI: color Doppler flow imaging
AUC: area under the curve
ROI: region of interest
LSCM: laser scanning confocal microscope
SD: Standard deviation

## References

[1] Wingo PA, Tong T, Bolden S (1995). Cancer statistics, 1995. CA Cancer J Clin.

[2] Rossi A, Maione P, Colantuoni G, Guerriero C, Ferrara C, Del GF, Nicolella D, Gridelli C (2005). Treatment of small cell lung cancer in the elderly. The Oncologist.

[3] Oremek GM, Sapoutzis N (2003). Pro-gastrin-releasing peptide (Pro-GRP), a tumor marker for small cell lung cancer. Anticancer Res.

[4] Molina R, Filella X, Augé JM (2004). ProGRP: a new biomarker for small cell lung cancer. Clin Biochem.

[5] Fan X, Wang L, Guo Y, Tu Z, Li L, Tong H, Yang X, Li R, Fang K (2015). Ultrasonic nanobubbles carring atti-PSMA nanobody: Construction and application in prostate cancer targeted imaging. PLoS One.

[6] Jiang Q, Hao S, Xiao X, Yao J, Bing O, Zhao Z, Liu F, Xin P, Luo B, Hui Z (2016). Production and characterization of a novel long-acting Herceptin-targeted nanobubble contrast agent specific for Her-2-positive breast cancers. Breast Cancer.

[7] Yang H, Cai W, Xu L, Lv X, Qiao Y, Li P, Wu H, Yang Y, Zhang L, Duan Y (2015). Nanobubble-Affibody: Novel ultrasound contrast agents for targeted molecular ultrasound imaging of tumor. Biomaterials.

[8] Gniazdowska E, Koźmiński P, Bańkowski K, Ochman P (2014). 99mTc-labeled vasopressin peptede as a radiopharmaceutical for small cell lung cancer (SCLC) diagnosis. Chem.

[9] Iochmann S, Lerondel S, Bléchet C, Lavergne M, Pesnel S, Sobilo J, Heuzé-Vourc’h N, Le Pape A, Reverdiau P (2012). Monitoring of tumour progression using bioluminescence imaging and computed tomography scanning in a nude mouse orthotopic model of human small cell lung cancer. Lung Cancer.

[10] Lapa C, Lückerath K, Rudelius M, Schmid JS, Schoene A, Schirbel A, Samnick S, Pelzer T, Buck AK, Kropf S (2016). [68Ga]Pentixafor-PET/CT for imaging of chemokine receptor 4 expression in small cell lung cancer - initial experience. Oncotarget.

[11] Yin T, Wang P, Zheng R, Zheng B, Cheng D, Zhang X, Shuai X (2012). Nanobubbles for enhanced ultrasound imaging of tumors. Int J Nanomedicine.

[12] Ushikubo FY, Furukawa T, Nakagawa R, Enari M, Makino Y, Kawagoe Y, Shiina T, Oshita S (2010). Evidence of the existence and the stability of nano-bubbles in water. Colloids & Surfaces A Physicochemical & Engineering Aspects.

[13] Deshpande N, Needles A Willmann JK (2010). Molecular ultrasound imaging: current status and future directions. Clin Radiol.

[14] Marxer EE, Brüssler J, Becker A, Schümmelfeder J, Schubert R, Nimsky C, Bakowsky U (2011). Development and characterization of new nanoscaled ultrasound active lipid dispersions as contrast agents. Eur J Pharm Biopharm.

[15] Rapoport N, Gao Z, Kennedy A (2007). Multifunctional nanoparticles for combining ultrasonic tumor imaging, targeted chemotherapy. J Natl Cancer Inst.

[16] Xing Z, Wang J, Ke H, Zhao B, Yue X, Dai Z, Liu J (2010). The fabrication of novel nanobubble ultrasound contrast agent for potential tumor imaging. Nanotechnology.

[17] Mcdonald DM, Baluk P (2002). Significance of blood vessel leakiness in cancer. Cancer Research.

[18] Stylianopoulos T, Jain RK (2013). Combining two strategies to improve perfusion and drug delivery in solid tumors. Proc Natl Acad Sci U S A.

